# Listen to Your Heart and Mind: A Pilot Mixed‐Methods Study on Combined Screening for Raised Blood Pressure, Atrial Fibrillation, Cognitive Impairment and Hearing Loss in Older Adults

**DOI:** 10.1111/ajag.70243

**Published:** 2026-10-07

**Authors:** Isabella Tan, Aletta E. Schutte, Catherine Morgan, Henry Brodaty, Sonali R. Gnanenthiran, Katya Numbers, Hueiming Liu, Menglu Ouyang, Paola Incerti, Padraig Kitterick, Mridula Sharma

**Affiliations:** ^1^ The George Institute for Global Health UNSW Sydney Sydney New South Wales Australia; ^2^ School of Population Health UNSW Sydney Sydney New South Wales Australia; ^3^ National Acoustic Laboratories Sydney New South Wales Australia; ^4^ Centre for Healthy Brain Ageing, Discipline of Psychiatry and Mental Health, School of Clinical Medicine UNSW Sydney Sydney New South Wales Australia; ^5^ Cardiology Department Concord Repatriation General Hospital Sydney New South Wales Australia; ^6^ Department of Linguistics Macquarie University Sydney New South Wales Australia; ^7^ Caring Futures Institute College of Health and Enablement Flinders University Adelaide Australia

**Keywords:** atrial fibrillation, cognition disorders, diagnostic screening programmes, hearing loss, hypertension

## Abstract

**Objective:**

To determine the potential feasibility and acceptability of combined screening for raised blood pressure (BP), atrial fibrillation (AF) and cognitive impairment (CI) in older adults within a setting of hearing health assessment for detection of hearing loss.

**Methods:**

We recruited participants aged 65 years or older (*n* = 35; mean age 73 ± 6 years, 51% females) from the community to attend a single visit lasting approximately 60–90 min. Prior to a comprehensive hearing assessment, BP was measured with an automatic BP monitor with AF detection, and CI screening was conducted using the patient section of the General Practitioner Assessment of Cognition instrument. A subset of participants (*n* = 16, 50% females) was interviewed post study to evaluate acceptability.

**Results:**

A total of 21 positive screens (six raised BP; two possible AF; 13 hearing loss) and 12 potential positive screens for possible CI were detected in 25 (71%) participants. Eight participants (23%) had more than one newly detected condition. Interviews revealed that combined screening was feasible and acceptable as participants felt it was ‘*convenient,*’ ‘*saves time*’ and ‘*more efficient*.’

**Conclusions:**

Combined screening for raised BP, AF and CI in older adults within a hearing health assessment setting appears feasible and was generally perceived positively by participants in this pilot study. Further implementation studies should be conducted with process and outcome evaluation embedded to confirm feasibility and acceptability as well as to evaluate cost‐effectiveness and health outcomes for combined screening in older adults.

## Practice Impact Statements

With appropriate training and planning, combined screening for raised blood pressure, atrial fibrillation and cognitive impairment in older adults may be integrated into audiology practice to improve the detection of these chronic conditions for early intervention and management.

## 1 Introduction

One in 6 (16%) Australians are aged 65 years and over. By 2066, an estimated 4.5 million Australians will be aged 65 years or over, increasing to just over 20% of the population [1]. Although people are living longer lives, many non‐communicable diseases increase in prevalence with age. Healthy ageing is thus an international health priority, prompting the launch of the United Nations' (UN) Decade of Healthy Ageing collaboration [2]. One key action in the UN Decade of Healthy Ageing is to ‘scale‐up primary health care to provide a comprehensive range of services for older people, including vaccination, screening, prevention, control and management of noncommunicable (including dementia) and communicable diseases…’ [3]^p17^.

The purpose of screening is to detect health problems in apparently healthy individuals, resulting in earlier treatment or intervention to reduce the risk of morbidity or mortality caused by such conditions [4]. For older adults, ischaemic heart disease, stroke and dementia are among the leading causes of death.

Raised blood pressure (BP) is the leading risk factor for both heart disease and stroke [5]. The prevalence of persistent, raised BP, clinically diagnosed as hypertension increases with age. However, half of those with raised BP are unaware of their condition due to the lack of associated symptoms, and one in five who are diagnosed with hypertension do not have their BP adequately controlled despite already being on treatment [5]. Hypertension also increases the risk of atrial fibrillation (AF) [6], a risk factor for stroke that primarily affects older adults and similarly often goes undetected [7]. Atrial fibrillation is itself associated with cognitive decline [8] and hearing loss [9], the latter of which has recently been established as one of the top modifiable risk factors for dementia and cognitive decline in adults in midlife (defined as age 18–65 years) and beyond [10].

Among Australians aged 65 years and over, around one in three has some degree of hearing loss [11], around two in three have raised BP [12]—many of whom would have co‐existing AF [13] and around one in five experiences cognitive impairment [14]. Early detection of these conditions through screening and subsequent treatment can result in reduced risk of stroke, heart disease and dementia and improved quality of life for the future. However, there is currently a lack of consistent, routine screening for these conditions. Furthermore, attending multiple visits to screen for different health conditions can be cumbersome and time‐consuming. Integrating screening testing for multiple chronic conditions into a health setting outside of general practice may improve the detection of underlying ‘silent’ conditions and allow for early intervention and treatment. As a large proportion of people who access hearing services are older adults, this pilot study aimed to determine the potential feasibility and acceptability of combined screening for raised BP, AF and cognitive impairment in older adults within the context of a hearing health assessment for detection of hearing loss.

## 2 Methods

### 2.1 Participant Recruitment

A convenience sample of 38 adults was recruited between September and December 2023 in metropolitan Sydney, Australia, through study advertisements posted in community newsletters, noticeboards (university and local library) and a secure, electronic research participant database (National Acoustic Laboratories, Sydney, Australia). The study was advertised as a ‘free, comprehensive hearing check, as well as a check for blood pressure, heart rhythm, and memory/thinking function.’ Thirty‐five participants completed the study. Participants were included if they were aged 65 years or older, able to give informed written consent and proficient in English. Exclusion criteria were self‐reported clinically diagnosed cognitive impairment, dementia or intellectual disability. Written informed consent was obtained from all participants. The study protocol was approved by the Human Research Ethics Committees of the University of New South Wales (HC230290), Macquarie University (520231590152778) and the National Acoustic Laboratories (HAHREC 2023‐23).

### 2.2 Study Protocol

Eligible participants attended a single appointment at Macquarie University's MQ Health Speech and Hearing clinic. Demographic information (age, sex, ethnicity, years of education) and self‐reported medical history were first collected through a short questionnaire. Participants then underwent screening for raised BP, AF and cognitive impairment prior to receiving a comprehensive hearing assessment (Figure 1). Detailed assessment procedures and criteria for a positive screen result for each condition are outlined in the Methods S1 and summarised in Figure 1. A positive screen result did not indicate a definitive diagnosis for any condition, and participants who received a positive screen result were advised to see their primary care physician for further testing and diagnosis. For cognitive impairment screening, as only the patient section of the two‐part screening instrument (General Practitioner Assessment of Cognition, GPCOG [15]) could be administered (see Methods S1), a positive screen result could not be confirmed. A result from the patient section of GPCOG warranting further investigation with the second part of the instrument was considered a potential positive screen.

**FIGURE 1:**
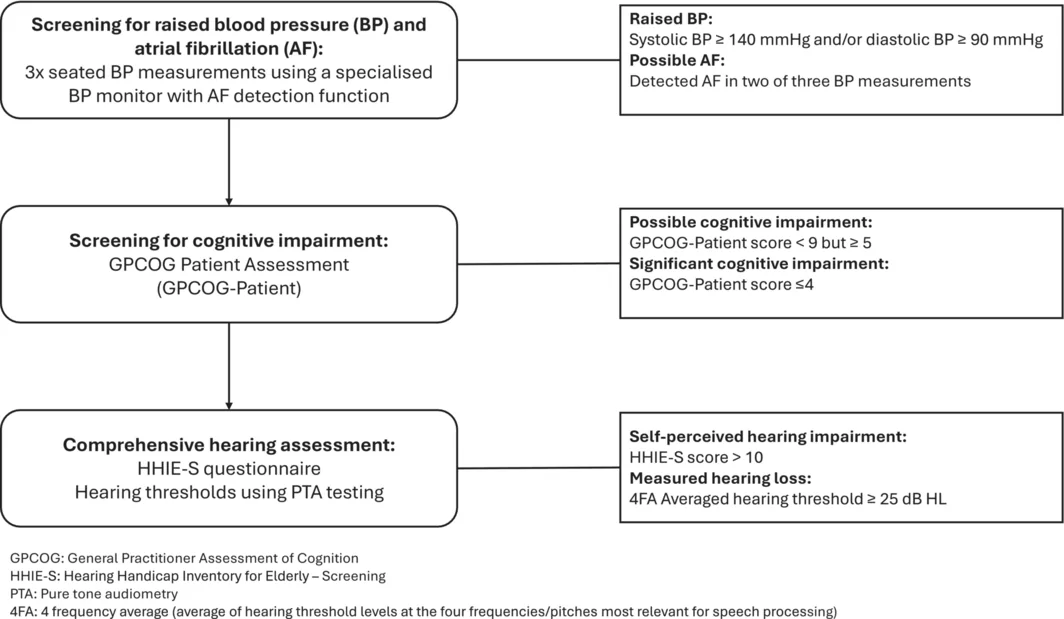
Workflow of combined screening and definitions of a positive screen for raised blood pressure, atrial fibrillation, cognitive impairment and hearing loss.

Two trained research staff administered screening for raised BP, AF and cognitive impairment. Hearing assessment was conducted by a qualified clinical audiologist or a final year student clinical audiologist under supervision of a qualified clinical audiologist (MS). A report of the screening test results and a letter to a general practitioner summarising the results were provided to all participants after the study. Total duration of the study appointments ranged from 60 to 90 min.

### 2.3 Participant Interviews

To determine the acceptability of the combined screening strategy, semi‐structured interviews were conducted with a subset of participants (interview guide provided in the Supporting Information S1). Interview questions were asked around three predefined domains: (1) Awareness or experience of screened conditions; (2) effectiveness of combined screening; (3) value proposition for combined screening. We used purposive sampling to invite participants for interviews with the aim to achieve an even distribution of age, sex, self‐reported hypertension status, measured BP, presence/absence of AF, presence/absence of possible cognitive impairment based on scores for the patient section of the GPCOG instrument (GPCOG‐Patient) and presence/absence of measured hearing loss.

Participants were asked for consent to be contacted for participation in the interview at the end of the study appointment. Eligible participants according to criteria stated above were then invited for the interview within 2 days of the study appointment. They were informed at time of invitation that the researchers were interested in understanding their experience of the combined health screening and how their views can improve the implementation of combined health screening more widely. All invited participants agreed to be interviewed.

Interviews were conducted over the phone by one researcher (IT) who received training in facilitating qualitative interviews from experts in process evaluation (HL and MO). Interviews were audio‐recorded with a computer using Voice Memos software (Apple Inc., Cupertino, USA) and transcribed by a professional transcription service. Most interviews were conducted one‐on‐one, but on two occasions, married couples were interviewed together. Interviews took place on average 7 days after the study appointment. All interviewed participants provided verbal informed consent. Duration of interviews was approximately 15 min.

### 2.4 Data Analysis

#### 2.4.1 Quantitative Analysis

Quantitative data are presented as mean ± standard deviation (SD) unless otherwise stated. Descriptive statistics were used to present participant characteristics and screening results (detected raised BP, possible AF, possible cognitive impairment and measured hearing loss).

#### 2.4.2 Qualitative Analysis

Interview transcripts were imported into NVivo software (NVivo Windows, release 1.7.2) and analysed by a single researcher (IT, who also conducted the interviews) with support from two senior researchers (HL and MO). A reflexive thematic analysis approach was used for the analysis of the interviews [16]. The researcher first familiarised themselves with the interview transcripts by reading the transcripts at least twice. Initial inductive coding was conducted, where preliminary codes were produced in relation to the domains explored in the interviews, and a stepwise process was used to develop the codebook (Table S1). Regular discussions with the two senior researchers and iterative review of the codebook helped enhance rigour and ensure transparency in coding decisions. The analytical rigour was enhanced through reflexive memo keeping, regular supervisory discussions and transparent documentation of the analytic process. Recurring themes were identified throughout the analysis in an iterative manner. Qualitative results are reported in line with the Consolidated criteria for Reporting Qualitative checklist (Table S2) [17].

## 3 Results

### 3.1 Participant Characteristics

Demographics and baseline characteristics of the participants (*n* = 35, mean age 72 ± 6 years, 51% females) are presented in Table 1. Twenty participants (57%) self‐reported having diagnosed hypertension, of which 3 (9%) also self‐reported having diagnosed AF. Eight participants (23%) self‐reported having hearing loss based on prior hearing screening or assessment, but only four wore hearing aids regularly.

**TABLE 1:** Participant characteristics and self‐reported medical history.

	Total (*n* = 35)	Male (*n* = 17)	Female (*n* = 18)
Age, (years)‐mean ± SD	72 ± 6	72 ± 7	72 ± 5
*Ethnicity (self‐reported)—n* (%)			
White	21 (60)	9 (53)	12 (67)
East/Southeast Asian	12 (34)	6 (35)	6 (33)
South Asian	1 (3)	1 (6)	0 (0)
Mixed	1 (3)	1 (6)	0 (0)
English as second language^b^—*n* (%)	13 (37)	6 (35)	7 (39)
*Education level—n* (%)			
Tertiary education or higher	28 (80)	14 (82)	14 (78)
Secondary education	7 (20)	3 (18)	4 (22)
*Self‐reported medical history—n* (%)			
Hypertension	20 (57)	11 (65)	9 (50)
Atrial fibrillation	3 (9)	3 (18)	0 (0)
Self‐reported hearing loss^a^	8 (23)	2 (12)	6 (33)
Regular hearing‐aid use	4 (11)	1 (6)	3 (17)
Hyperlipidaemia	19 (54)	6 (35)	13 (72)
Type 2 diabetes	6 (17)	5 (29)	1 (6)
Coronary artery disease	3 (9)	3 (18)	0 (0)
Bradycardia	1 (3)	1 (6)	0 (0)
Previous stroke	2 (6)	2 (12)	0 (0)
Previous heart attack	2 (6)	2 (12)	0 (0)
Chronic kidney disease	1 (3)	1 (6)	0 (0)
Cancer	12 (34)	6 (35)	6 (33)
Sleep apnoea	9 (26)	6 (35)	3 (17)

^a^ Self‐reported hearing loss is determined as whether a participant has been informed by a professional audiologist that he/she has hearing loss.

^b^ English as a second language is determined if the language spoken by an individual as a child is a language other than English.

### 3.2 Detection of Raised Blood Pressure, Atrial Fibrillation, Cognitive Impairment and Hearing Loss

Based on measured outcomes (Table S3), there were 21 positive screen results in 13 (37%) participants across conditions of raised BP, possible AF and hearing loss, whereby eight positive screens identified a newly detected condition in those participants (Table 2).

**TABLE 2:** Detection of raised blood pressure, atrial fibrillation, cognitive impairment and hearing loss based on measured outcomes.

Detected condition	Total (*n* = 35)	Male (*n* = 17)	Female (*n* = 18)
	*n* (%)	*n* (%)	*n* (%)
Raised BP (i.e., measured BP ≥ 140/90 mmHg)	6 (17)	3 (18)	3 (17)
Newly detected	2 (6)	1 (6)	1 (6)
Possible atrial fibrillation	2 (6)	1 (6)	1 (6)
Newly detected	1 (3)	0 (0)	1 (6)
Possible cognitive impairment (GPCOG‐Patient score of 5–8)^a^	12 (34)	7 (41)	5 (28)
Hearing loss (i.e., 4FA ≥ 25 dB HL in either or both ears)	13 (37)	6 (35)	7 (39)
Newly detected	5 (14)	4 (24)	1 (6)
More than one condition detected	8 (23)	4 (24)	4 (22)
Raised BP and possible cognitive impairment	1 (3)	1 (6)	0 (0)
Raised BP and hearing loss	2 (6)	0 (0)	2 (11)
Possible atrial fibrillation and possible cognitive impairment	2 (6)	1 (6)	1 (6)
Possible cognitive impairment and hearing loss	3 (9)	2 (12)	1 (6)

*Note:* 4FA: average hearing threshold across four frequencies 500 Hz, 1 kHz, 2 kHz, 4 kHz.

Abbreviations: BP, blood pressure; GPCOG, General Practitioner assessment of Cognition.

^a^ No participants had GPCOG‐Patient score ≤ 4.

Of the six participants (17%) with measured raised BP, two were previously unaware and four were already on treatment for hypertension. Possible AF was detected in two participants, one of whom was previously undiagnosed.

Twenty‐three participants (66%) achieved the maximum score for GPCOG‐Patient, indicating no cognitive impairment. Twelve participants (34%) had a score that potentially indicated possible cognitive impairment, but a positive screen could not be confirmed due to the absence of informant interviews. These participants were unable to complete one or more of the following tasks: provide the correct date (*n* = 3), clock drawing (*n* = 4) and recall components of the name and address information they were asked to remember at the start of the GPCOG test (*n* = 5). No participants had a score that indicated significant cognitive impairment.

Of the 13 participants who had measured hearing loss (37%), four did not report self‐perceived hearing handicap. Importantly, 33 of 35 participants (94%) had impaired hearing at very high frequencies (i.e., > 8 kHz). These frequencies are associated with speech perception in noisy environments [18] and hearing impairment at these frequencies and may be a precursor to future hearing loss [19].

### 3.3 Detection of Multiple Conditions

Excluding potential positive screen of possible cognitive impairment, eight participants (23%) had more than one condition detected through combined screening (Table 2). When considering existing (self‐reported) conditions, 19 participants (54%) had more than one of the screened conditions (Table S4).

### 3.4 Qualitative Findings

Characteristics of the 16 participants (nine females, mean age 74 ± 6 years) interviewed and not interviewed are shown in Table 3. The identified themes in relation to feasibility and acceptability of combined screening with illustrative quotes are presented in Table 4. A summary of the findings is detailed below with theme numbers identified in parentheses.

**TABLE 3:** Characteristics of interviewed participants and those not interviewed.

	Interviewed (*n* = 16)	Not interviewed (*n* = 19)
Age, (years)	74 ± 6	70 ± 6
Females, *n* (%)	9 (56)	9 (47)
Measured raised blood pressure, *n* (%)	3 (19)	3 (16)
Self‐reported hypertension, *n* (%)	10 (63)	10 (53)
Detected atrial fibrillation, *n* (%)	1 (6)	0 (0)
Self‐reported atrial fibrillation, *n* (%)	1 (6)	2 (11)
GPCOG‐Patient score < 9, *n* (%)	7 (44)	5 (26)
Measured hearing loss, *n* (%)	7 (44)	6 (32)

Abbreviation: GPCOG, general practitioner assessment of cognition.

**TABLE 4:** Selected quotes from participants for each domain/theme.

Domain/theme	Illustrative quotes
1. Awareness and experience of screened conditions	
1.1 General awareness/knowledge about hypertension	Aware I have hypertension, also family history, mother died of heart failure, son of heart attack, brother had stroke and recovered, sister died of heart attack, so very aware of my heart condition.—Participant 2 I know it can be a serious consequence.—Participant 8 I know you don't necessarily have to feel unwell to have high blood pressure… I'm aware of why I have high blood pressure, because I have an oversized heart and its caused issues for me all my life.—Participant 13
1.2 Limited awareness/knowledge of atrial fibrillation	I know that [atrial fibrillation] is sort of a change in the frequency of your heartbeats. I do know a couple of people who have got that condition, so I don't know a lot about it, but I know that there's a change in the frequency of your heartbeat.—Participant 10 Yeah, I watch a few medical shows… Well, it's when a defibrillator is used for if you have extreme [for that] condition, they have to sort it out for you or you'll die. Am I right?—Participant 14
1.3 Awareness/experience of hearing impairment observed by self and/or others	I think it may ‐ Ive lost a little bit of hearing in the last few years… Other people are telling me or have to repeat themselves, yes, I do tend to notice it more then.—Participant 19 To some extent, yes. I have a tendency now to put the text onto the television… Also, then the problem of hearing conversations in restaurants or in noisy places.—Participant 29
1.4 Awareness of cognitive changes observed as short‐term memory lapses	I have to admit that from time to time, as Ive got older – and some of my friends have this problem as well – when you're trying to think of the name of somebody in a TV series that you've watched and you would say, oh yeah, [it was] a really great show, you can describe it in detail, you think, oh, I can't remember the name of the character, or something like that.—Participant 10 Sometimes I can find it difficult to come up with a name or something I know is in the back of my mind.—Participant 29 Things like I put down something and I come back [unclear to pick it up]. I can't remember where I placed it.—Participant 8 You can go from one room to another; you forget what you were supposed to do. You can't remember what you ate for breakfast.—Participant 20
1.5 Unequal levels of concern/worry across conditions	More concerned about cognitive function. Other conditions can compensate, BP can monitor and take medication, yearly review with cardiologist, hearing can use hearing aids, but think cognitive decline will impact life more and there is nothing I can do to compensate for the decline.—Participant 2 Any cognitive decline would worry me. Then hearing would be a problem too, because if you can't hear properly, then you can't participate properly in conversations. I read something lately where they say there might be a link between the loss of hearing and cognitive decline, so – yes.—Participant 10 Hearing is bad ‐ loss of hearing is bad and I suppose the cognitive, getting dementia is bad as well, but probably not as bad as having a stroke or having a heart attack. That's where I put them in the ‐ if they could go in order, in terms of severity of need for attention would be, blood pressure followed by cognitive understanding followed by hearing.—Participant 13 I think the high blood pressure is probably more important, to me, anyway. Because high blood pressure, you don't know, really, unless you measure.—Participant 21 I think atrial fibrillation would be more concerning. My brother suffered from atrial fibrillation, and I'm aware of the impact that it had on his day‐to‐day activities.—Participant 30
2. Effectiveness of combined screening on raising awareness and/or changed attitudes and/or behaviour	
2.1 No change in attitude towards raised BP	Will continue to monitor BP due to family history. Participation in the study did not change my views. Important to monitor BP as per before.—Participant 2 When I go to see the GP, if I have some condition, things like fever or something like that, I think the GP will check the heart pressure whenever I visit. I don't think I will consider measuring my blood pressure unless there's a [really special] condition happen to me.—Participant 8
2.2 Increased awareness of importance of other conditions	Atrial fibrillation is also a concern, I guess, now I know what it is.—Participant 14 I'm more aware of degeneration of thinking and cognitive functioning.—Participant 19 I guess, I learned from my visit yesterday, it was emphasised I should be having my hearing checked once a year which I haven't done. If I start doing that as advised, then ‐ and I accept that that's a sensible thing to do, because I go and have my eyes tested but I don't go and have my ears tested.—Participant 29
3. Value proposition of combined health screening	
3.1 Doing tests together is convenient and saves time	Well, you know the outcome of three things at one appointment, and I think it's more efficient.—Participant 9 I don't want to spend my life going from doctor to doctor if I don't need to. Yeah, so I think the saving the time in getting it all done in one go.—Participant 10 Just easier to do it all in one go, more convenient, yeah.—Participant 26
3.2 Perceived benefits and barriers to adding tests to specific health appointments	Well, my view about this is it's a bit like a paralegal. The big worry is, are the people qualified enough to do the tests… It's this confidence issue.—Participant 17 Well, from my point of view, I would – you're usually offered these things, and you take them on, or you reject them, right? With me, if I'm concerned about something, like I did the Specsavers test for the hearing because I was concerned about my hearing. I didn't know whether they were credible to give you that sort of information, but I thought it was better than nothing… But if someone was going to ask me to do a blood pressure thing, then Id probably not be interested.—Participant 18 I think the advantage would be to get it all done in one go [than] be having to – as I said before, not having to trot off to all separate places. I mean, I would assume that it would be a qualified audiologist that would be testing your hearing, and it would be a doctor or someone with some medical experience testing your blood pressure, et cetera.—Participant 10 In general, Id happy to say, yeah, well why not check the other things at the same time. But then, on the other hand, Id also think, well no, am I being – am I having services pushed onto me which I don't really need, or want? But no, I think in general, yeah, why not?—Participant 30
3.3 Importance of follow‐up action post screening	It will be better for someone, yeah, to be more sure whether the heart does have a problem and they [detect it], they go ahead to do another further study, something like that. I think it's better to do that.—Participant 7 It would depend on how, then, information… is communicated, say, would there be an automatic letter sent to the GP or somebody that's got the oversight of your health needs to be linked in to the results.— Participant 29
3.4 Concerns regarding screening test results	I'm not sure that too many people would welcome the dementia test…because people have a great fear of it, if theyre like me which I think they might be… I think a lot of people just wouldn't want to know if their cognitive function was declining.—Participant 14 [The study] was short and sweet, but it put my mind at ease, particularly with, well – and two things. One is my mind was put at ease by the fact that my hearing loss wasn't outside my age bracket. The fact that I slumped the other one, that is a real worry for me, and I don't really want to go into the Alzheimer's – I would be very interested in pursuing that further, but saying that, I would also be very scared to do that because I wouldn't want to really completely confront it with the future, if you know what I mean?—Participant 18

#### 3.4.1 Domain: Awareness and/or Experience of Screened Conditions

This domain sought to understand participants' general awareness and lived experience of each of the screened conditions to gauge acceptability of combined screening. Overall, participants demonstrated general awareness about hypertension (1.1) but limited awareness of AF (1.2). All interviewed participants reportedly noticed cognitive changes, particularly short‐term memory lapses or forgetting names (1.4). Most attributed these changes to *‘senior moments’* and *‘*part of growing old.’ All interviewed participants who had measured hearing loss during the screening reported subjective awareness of hearing impairment (1.3).

When asked whether raised BP, AF, cognitive impairment or hearing loss would be more concerning, participants indicated varying levels of concern across the conditions (1.5). Despite not being concerned about self‐reported short‐term memory lapses, participants (*n* = 8) were most worried about cognitive impairment (and by association, dementia), citing lack of perceived treatment and the lived experience of caring for someone with dementia being the major drivers for concern. Some were more concerned about raised BP and AF due to the consequences of heart attack and stroke, while others were worried about the impact of hearing loss leading to social isolation and possibly dementia.

#### 3.4.2 Domain: Effectiveness of Combined Screening

In general, there was no change in the participants' attitude towards raised BP—those who already had diagnosed hypertension indicated they would continue monitoring their own BP, either at home or at their doctor's clinic, and those who did not have measured raised BP did not feel the need to monitor more regularly than at the doctor's clinic (2.1). However, one participant with hypertension who unexpectedly had measured raised BP was motivated to purchase a new BP device to monitor BP more regularly.

Combined screening increased awareness of AF for those who previously have not heard of the condition or had only come across the term in passing but did not understand it (2.2). Some participants indicated being more aware of cognitive changes and importance of regular hearing checks as a result of the combined screening (2.2).

#### 3.4.3 Domain: Value Proposition of Combined Health Screening

All interviewed participants were unanimous in their support of combined screening in a single visit. Reasons cited included efficiency, convenience and the time‐saving nature of having all tests performed together (3.1). One interviewee also noted the value of multidisciplinary teams with combined screening.

Most interviewed participants reported the overall study duration was acceptable, though two participants reported the length of the hearing assessment component (approximately 45 min) was slightly onerous.

When combined screening was proposed as an add‐on to existing health appointments, the responses from interviewees were more mixed. While some readily thought it would be a good thing, others noted concerns about whether additional tests would be pushed on to people unnecessarily, and whether staff would be qualified to conduct the tests and therefore whether the screening results would be deemed trustworthy (3.2).

Several participants noted the importance of following up when a health condition is identified from screening, as well as communicating screening results to their doctor (3.3).

Some participants raised concerns regarding cognitive screening, noting a fear of implications from a result indicative of cognitive impairment (3.4).

## 4 Discussion

This pilot study demonstrated that combined screening for raised BP, AF and cognitive impairment in older adults within the setting of hearing health assessment for detection of hearing loss was potentially feasible and acceptable. Excluding possible cognitive impairment, there were eight new detections across the conditions of raised BP, possible AF and hearing loss. Qualitative interviews revealed that combined screening was generally perceived positively and of value to participants but highlighted possible challenges in terms of workforce and post‐screening implications.

### 4.1 Current Screening Recommendations in Older Adults

Screening recommendations for raised BP, AF, cognitive impairment and hearing loss in older adults differ by countries.

Annual BP screening by a general physician is recommended for all adults aged 18 years or older in the United States [20], and every 2–5 years in Europe [21] and Australia [22] based on age and cardiovascular risk. For adults in Australia aged 45–79 years without known cardiovascular disease (CVD), BP screening is included as part of the recommendation to assess risk using the Australian CVD risk calculator [22].

Guidelines on screening for AF, hearing loss and dementia are limited, despite known benefits of early detection and treatment of these conditions, due to insufficient evidence to assess balance of benefits and harms of screening [23, 24, 25]. Opportunistic AF screening in adults aged 65 years or older has been recommended in Australia [26], and routine assessment of heart rhythm in those aged 65 years or older has been recommended in Europe [27]. Although current guidelines cite insufficient evidence to recommend screening of hearing loss [25] and cognitive impairment [24] in asymptomatic older adults, opportunistic case finding is still supported.

### 4.2 Feasibility and Acceptability of Combined Screening

In terms of workforce feasibility within the context of the present study, screening for raised BP and AF could be performed by non‐health professionals who are trained in performing a BP measurement using a specialised automated BP monitor [28]. Cognitive assessment using the GPCOG screening tool could also be conducted by a trained non‐clinician, as was done in the present study. As such, integrating combined screening for raised BP, AF and cognitive impairment in older adults into the audiology setting may be feasible and can be successfully conducted by trained practice staff. However, conducting the second stage of the GPCOG instrument may not be practical within the audiology setting as it may require conducting informant interviews on a separate day to the screening. Other brief cognitive impairment screening instruments, such as the Standardised Mini‐Mental State Examination (which takes approximately 5–10 min) [29], should be considered for future implementation of combined screening.

In terms of time acceptability, combined screening of the raised BP, AF and cognitive impairment within a hearing health assessment, inclusive of a health questionnaire and a hearing screening survey, required 60–90 min. All interviewed participants found this duration acceptable, although some noted the hearing assessment was slightly onerous. The aim of the present study was to investigate the potential feasibility and acceptability of combined screening in the context of a hearing health assessment, which is why a comprehensive hearing check was performed.

While many participants remained supportive of implementing combined screening through providing add‐on tests to a specific health appointment (e.g., adding BP check at a hearing appointment), others were concerned about the implications on perceived pressure to undergo additional tests and the uncertainty of whether a qualified person would perform the tests. These responses highlight the importance of trust between health service providers and those to whom service is provided. Studies have shown a small to moderate correlation between trust and health outcomes, and between trust and health behaviours [30]. Some interviewees also noted the possibility of anxiety associated with a positive screen for a health condition, particularly for cognitive impairment. This emphasises the need to consider the benefits and harm of health screening in general [4]. In the context of cognitive screening, there can be direct or indirect harms caused by false‐positive results, which can result in negative emotions, such as anxiety, and fear of stigma [24]. In general, false‐negative results may give a false sense of assurance and delay diagnosis, but current evidence on potential harm of false‐negative screening results is limited [31].

Outside of the general practice setting, combined health screening is limited apart from through sporadic community screening campaigns. Examples include May Measurement Month, a global BP screening campaign that combined BP and AF screening in some countries including Australia [32], and mobile health screening services or workplace health checks that may include screening for cardiovascular risk factors as well as hearing loss. Whilst it has been suggested that audiological practice is well positioned for cognitive screening [33], it is unlikely that this has been implemented in practice.

### 4.3 Value of Combined Screening

The present study identified one in five individuals with one or more potential risk factors of heart disease, stroke, and dementia who may not otherwise have known about their health condition. Early detection with appropriate follow‐up diagnosis and treatment can significantly reduce the risks associated with raised BP, AF, cognitive impairment and hearing loss—conditions that predominantly affect older adults.

Although interviews revealed the present study did not change attitudes nor behaviours of participants toward raised BP, participation in the study increased their knowledge and awareness of AF, thinking and memory function deterioration and recognition of the importance of regular hearing checks. One participant emailed the research team post‐study that she indeed followed up with a specialist after a positive screen for possible AF, and while a benign irregular heart rhythm rather than AF was confirmed, she felt it was good to get everything checked out. Taken together, the study demonstrated that combined health screening would not only be effective in identifying people at risk but also has a role in health promotion and health education. However, knowledge alone is insufficient to effect change in behaviour. Participants also noted the importance of follow‐up action to screening results and ensuring results are effectively communicated to an individual's treating physician. Screening without a follow‐up on positive screen results and subsequent linkage to care would render the screening ineffective. Future implementation of combined health screening should include a robust linkage to care pathway to ensure positive screen results are followed up to confirm diagnosis and initiate treatment.

### 4.4 Limitations

The present study had several limitations that preclude the findings from being extrapolated to the general older population. As a pilot study, it was limited by the small sample size, and therefore, the study cohort may not be representative of the general Australian older adult population. Potential self‐selection bias may have been present due to the participants' higher than average education, which may imply higher health literacy and health engagement than the general older adult community. This may have diminished the rate of positive screens for raised BP and also skewed the qualitative feedback on acceptability of combined screening. Furthermore, one researcher (IT) was personally known to some of the interviewed participants (*n* = 7, 44%) through family or social connections. This may have influenced participants' responses, particularly regarding perceived acceptability of the screening approach. Although participants provided both positive and negative feedback regarding the screening experience, the potential for social desirability bias cannot be excluded. Consequently, the findings of the present study are not definitive, and findings related to acceptability should be interpreted cautiously and viewed as preliminary. However, the study findings can be used to inform the design and implementation of future studies on combined screening. Future studies should seek to involve larger, more representative samples and engage independent interviewers to further evaluate feasibility and acceptability of combined screening across diverse older adult populations.

Another limitation is that this study did not collect data to assess the effectiveness of combined screening in terms of follow‐up of positive screen results, nor did it assess cost‐effectiveness, which is an important factor for consideration before wider implementation of combined screening can occur. Future feasibility studies on combined screening should include collection of a follow‐up metric to determine downstream effectiveness of the screening.

In terms of detection of possible cognitive impairment, the pilot study was limited in its present findings as only the patient part of the GPCOG screening instrument was conducted, meaning that possible cognitive impairment could not be confirmed. The proportion of potential positive screens would therefore likely have more false positives than if the informant interviews were also conducted [15]. However, the findings nonetheless demonstrated that it is potentially feasible to conduct screening for possible cognitive impairment in combination with screening for other chronic conditions in the context of a hearing health assessment.

Lastly, the recall period between screening and interviews (average of 7 days) may introduce recall bias, especially in those with potential cognitive impairment. However, only two of the interview questions required specific recall of experience from the screening appointment. As such, recall bias was unlikely to have impacted participants' views of combined screening.

## 5 Conclusions

The present study demonstrates that combined screening for raised BP, AF and cognitive impairment in older adults is potentially feasible and acceptable within the setting of hearing health assessments. Further implementation research is warranted to determine the best strategies and settings beyond the audiology setting for combined health screening in older adults.

## Funding

This work was supported by the University of New South Wales's Cardiac, Vascular and Metabolic Medicine (CVMM) Theme Collaborative Grant.

## Ethics Statement

The study protocol was approved by the Human Research Ethics Committees of the University of New South Wales (HC230290), Macquarie University (520231590152778) and the National Acoustic Laboratories (HAHREC 2023‐23).

## Conflicts of Interest

A.E.S. received speaker honoraria and/or consultancy fees from Servier, Abbott, Sanofi, AstraZeneca, Medtronic, Omron, Aktiia, SiSU Health, Roche/Alnylam and Sky Labs. In the last 3 years, H.B. is or has been an advisory board member, consultant and/or speaker for Eisai, Eli Lilly, Medicines Australia and Skin2Neuron.

## Supporting information


**Table S1:** Codebook developed for qualitative analysis of interview transcripts.
**Table S2:** COREQ (COnsolidated criteria for REporting Qualitative research) checklist.
**Table S3:** Measured blood pressure, GPCOG scores, HHIE‐S scores and pure tone audiometry results from study cohort.
**Table S4:** Prevalence of concomitant conditions of raised blood pressure or hypertension, atrial fibrillation, possible cognitive impairment and hearing loss.

## Data Availability

The data underlying this article cannot be shared publicly nor privately as the ethical approval does not cover secondary use nor sharing of the collected data.
