# Supplementary material for: Listen to Your Heart and Mind: A Pilot Mixed‐Methods Study on Combined Screening for Raised Blood Pressure, Atrial Fibrillation, Cognitive Impairment and Hearing Loss in Older Adults
Source: Australas J Ageing. 2026 Oct 7;45(4):e70243. doi: 10.1111/ajag.70243 (PMC13643817; doi:10.1111/ajag.70243)
Supplement: Supplementary file 1 [file AJAG-45-0-s001.docx]

**Listen to your heart and mind: A pilot mixed-methods study on combined screening for raised blood pressure, atrial fibrillation, cognitive impairment and hearing loss in older adults**

**Supporting Information**

Contents

[Supplementary Methods	2](#_Toc236246275)

[Detailed screening procedures	2](#_Toc236246276)

[Interview guide	5](#_Toc236246277)

[Supplementary Tables	7](#_Toc236246278)

[Table S1. Codebook developed for qualitative analysis of interview transcripts.	7](#_Toc236246279)

[Table S2. COREQ (COnsolidated criteria for REporting Qualitative research) Checklist	9](#_Toc236246280)

[Table S3. Measured blood pressure, GPCOG scores, HHIE-S scores and pure tone audiometry results from study cohort.	11](#_Toc236246281)

[Table S4. Prevalence of concomitant conditions of raised blood pressure or hypertension, atrial fibrillation, possible cognitive impairment and hearing loss.	12](#_Toc236246282)

[References	13](#_Toc236246283)

**Supplementary Methods**

**Detailed screening procedures**

***Screening for raised blood pressure and atrial fibrillation***

After three minutes of quiet, seated rest, blood pressure (BP) was measured in triplicate on the participant’s non-dominant arm with 30-second intervals. A specialised automated BP monitor with atrial fibrillation (AF) detection (Microlife Watch BP Office 2G,^1^ Microlife Corporation, Taiwan) was used and BP measurements were performed in the presence of a research staff member (i.e. attended automated BP). A positive screen result for raised BP was recorded if the average of the second and third BP readings was either ≥ 140 mmHg for systolic BP, ≥ 90 mmHg for diastolic BP, or both.

Presence of possible AF was automatically determined by the BP monitor’s AF detection algorithm based on the standard deviation of heart rate over the measurement period (> 25% of average heart rate) during each BP measurement (with reported sensitivity of 95% and specificity of 86%),^2^ allowing for simultaneous BP and AF screening. For the purposes of this study, a positive screen result for possible AF was recorded if AF was detected in at least two of the three BP measurements.^2^

***Screening for cognitive impairment***

After BP measurements, participants underwent cognitive impairment screening using the first part of the General Practitioner Assessment of Cognition (GPCOG) instrument (GPCOG-Patient).^3,4^ The instrument consisted of five questions with nine cognitive test items including time orientation (1 point), clock drawing (2 points), reporting a recent event (1 point) and a memory recall task (5 points). For the memory recall task, participants were given a name (first name and surname) and street address (street number, street name, and suburb) at the beginning of the test, then asked to recall the information at the end. A GPCOG-Patient score of 9 indicates no cognitive impairment and a score of 4 or below suggests cognitive impairment. A score between 5 and 8 indicates possible cognitive impairment but the second part of the GPCOG tool (informant interview) should be administered to gain more information. If a participant scores four points or below, significant cognitive impairment is indicated, and informant interview using the GPCOG tool may not be necessary.

The GPCOG instrument was chosen for this study as the time required to administer the patient part was significantly shorter than other available instruments. However, due to the lack of scope for follow-up in this study, informant interviews were not conducted for those scoring between 5 and 8 in GPCOG-Patient, as it would require contacting an individual who knows the participant well outside of the screening appointment.

***Comprehensive hearing assessment***

A comprehensive hearing assessment was conducted in a separate quiet room adjacent to the room for the other screening tests. The ambient sound pressure level in the testing room was 29±4 dB SPL with dBA weighting. The ambient sound pressure level in the testing room was 29±4 dB SPL with dBA weighting.

The hearing assessment comprised the Hearing Handicap Inventory for Elderly – Screening (HHIE-S) questionnaire, hearing sensitivity assessment using pure tone audiometry (PTA) with extended high frequencies (250 Hz, 500 Hz, 1 kHz, 2 kHz, 4 kHz, 8 kHz, 9 kHz, 10 kHz, 11.2 kHz and 12.5 kHz), and physical examination of the external auditory canals and tympanic membranes using otoscopy and tympanometry.

The HHIE-S is a 10-item questionnaire that assesses a person’s subjective appraisal of the social and emotional impact of hearing loss. It is scored out of a maximum of 40 points, where 0 is no perceived handicap and 40 is maximum perceived handicap. A score of 10 or more may indicate hearing impairment.

PTA testing was performed with air conduction in both ears. Bone conduction on the better ear for clinical audiometry frequencies at 500 Hz, 1 kHz, 2 kHz and 4kHz was also performed. PTA identifies the hearing threshold levels at predefined frequencies (pitches), where higher thresholds indicate worse hearing. Measured hearing loss was defined if the averaged hearing threshold over the four frequencies most relevant for speech processing (4FA) was > 25 dB HL in either or both ears.^5^

Otoscopic examination was performed to visually inspect the ear canal and tympanic membrane for signs of disease and check for foreign objects and wax occlusion. Tympanometry is an objective test for evaluating middle-ear function.

## Interview guide

**LISTEN TO YOUR HEART AND MIND: Interview Guide**

1. **Introductory script**

Hello, my name is __________ (introduce self) and I am from _______ (explain role). Thank you for agreeing to participate in this interview for the Listen to Your Heart and Mind project. The project’s aim is to trial screening for high blood pressure, atrial fibrillation, and cognitive impairment at a hearing test appointment, as well as to determine the acceptability and feasibility of this additional screening. Therefore, the interview questions will ask about your experience in taking part in this project, as well as your awareness or experience with these health conditions. Your views and answers will help us understand ways to improve future implementations of combined health screening at hearing test appointments. The participant information statement provided a full description of the project to you.

The interview will take up to **30** minutes to complete, and with your permission, I would like to audio record the interview.

It is not anticipated that the interview will induce feelings of distress. However, if this occurs, you can stop the interview, take a break, or skip any questions you do not want to answer.

Before we proceed, do you have any questions?

- Yes, answer questions.
- No, proceed.

2. **Interview questions**

**Domain: Conditions of high blood pressure, cognitive impairment, atrial fibrillation and hearing loss**

How often do you monitor your blood pressure at home? What motivated you to have your blood pressure checked at home? How did you find the ease of doing it? Any examples of when you found it difficult?

Have you experienced any trouble with recalling past experiences or remembering things? Could you provide an example?

Have you experienced any trouble with hearing? Could you provide an example?

Are you aware of a condition called atrial fibrillation? What do you know about it?

Would you say you are equally concerned about having high blood pressure, hearing loss, or cognitive impairment? Do you think any of these conditions impact life more than the others?


**Domain: Effectiveness of Screening** (whether screening would change thoughts/behaviour)


How likely do you think you will get your blood pressure checked in the future (even if you don’t feel unwell)? Would your answer be different if you had not participated in this study?

How important do you think it is to monitor your blood pressure going forward? What about monitoring your memory or thinking function?

**Domain: Value proposition**

What are your thoughts about the duration of the study appointment?

If you were given a choice, what would you prefer—getting screened for high blood pressure and cognitive impairment at your hearing assessment appointment, or going to separate screening appointments for each of those? Why?

What advantages do you think there are in adding additional tests such as blood pressure and cognitive function screening to a health appointment that is specific to, for example in this study, hearing? What are the disadvantages?

 **Concluding questions:**

Is there anything else you would like to tell me that we have not spoken about in this interview?

**4. Interview closure script**

Thank you for your time today. It is very much appreciated, and your answers will no doubt be very helpful for determining how we consider integrated health screening in the future.

**Supplementary Tables**

**Table S1:** Codebook developed for qualitative analysis of interview transcripts

| **Name** | **Description** |
| --- | --- |
| Awareness of or experience with health conditions | This code captures a participant’s baseline awareness or lived experience of screened conditions (including hearing loss). |
| Atrial fibrillation | This child code captures a participant’s prior knowledge or experience with atrial fibrillation. |
| Hearing problems | This child code captures a participant’s prior experience with hearing issues, whether self-observed or observed by others. |
| High blood pressure | This child code captures a participant’s prior knowledge or experience with raised blood pressure. |
| Attitude toward self-monitoring of blood pressure | This subcode captures a participant’s attitude toward monitoring blood pressure at home. |
| Memory or thinking problems | This child code captures a participant’s observations (or awareness) regarding memory or thinking problems. |
| Concern about health condition | This code captures a participant’s relative level of concern across the screened conditions. |
| Equal concern | This child code captures equal levels of concern across the screened conditions, that is, a participant feels all conditions are important. |
| One more than the other | This child code captures a participant’s perception of one or more condition being of more concern than the others. |
| Coping mechanisms | This code captures a participant’s reported coping mechanisms in experiencing any of the screened conditions (including hearing loss). |
| Effectiveness of screening | This code captures whether there were any changes in knowledge, attitude of behaviour to any of the screened conditions as a result of participation in the pilot study. |
| Changed attitude to any one or more health condition | This child code captures a participant’s changed attitude/knowledge/behaviour to any one of more of the screened conditions. |
| No change | This child code captures instances where a participant indicated participation in the screening did not result in any changes in relation to the screened conditions. |
| It’s part of growing old. | This code captures a participant’s attitude toward screened conditions as related to ageing. |
| Miscellaneous | This code is used to capture any comments that may not fit in the other codes. |
| Perceived impact of health condition | This code captures a participant’s relative perception of the impact of the screened conditions on quality of life. |
| Equal impact | This child code captures equal levels of impact on quality of life across the screened conditions i.e. a participant feels all conditions impact quality of life equally. |
| One more than the other | This child code captures a participant’s perception of one or more condition having more impact on the quality of life than the others. |
| Value proposition | This code captures a participant’s perceived value of combined screening for raised blood pressure, atrial fibrillation, cognitive impairment and hearing loss. |
| Adding tests to condition-focused appointment | This child code captures a participant’s perception of value of adding screening tests to a health appointment that was meant for a specific health condition (e.g. adding the tests to an optometry appointment or audiology appointment). |
| Having a single appointment for all conditions | This child code captures a participant’s perception of value of having a single appointment to screen for multiple health conditions (raised blood pressure, atrial fibrillation, cognitive impairment and hearing loss). |
| Negatives | This child code captures any negative sentiment a participant expressed toward combined screening. |
| Other suggestions | This child code captures any suggestions from participants on how combined screening could be improved or on suggestions to take into consideration for future implementation of combined screening. |
| Positives | This child code captures any positive sentiment a participant expressed toward combined screening. |

**Table S2:** COREQ (COnsolidated criteria for REporting Qualitative research) Checklist

| **Topic** | **Item No.** | **Guide Questions/Description** | **Reported on**  **Page No.** |
| --- | --- | --- | --- |
| **Domain 1: Research team**  **and reﬂexivity** | | | |
| *Personal characteristics* | | | |
| Interviewer/facilitator | 1 | Which author/s conducted the interview or focus group? | 9 |
| Credentials | 2 | What were the researcher’s credentials? e.g. PhD, MD | 9 |
| Occupation | 3 | What was their occupation at the time of the study? | 9 |
| Gender | 4 | Was the researcher male or female? | 9 |
| Experience and training | 5 | What experience or training did the researcher have? | 9 |
| *Relationship with*  *participants* | | | |
| Relationship established | 6 | Was a relationship established prior to study commencement? | 22 |
| Participant knowledge of  the interviewer | 7 | What did the participants know about the researcher? e.g. personal goals, reasons for doing the research |  |
|  |  |  | 9 |
|  |  |  |  |
| Interviewer characteristics | 8 | What characteristics were reported about the interviewer/facilitator? e.g. Bias, assumptions, reasons and interests in the research topic |  |
|  |  |  | 22 |
|  |  |  |  |
| **Domain 2: Study design** | | | |
| *Theoretical framework* | | | |
| Methodological orientation and Theory | 9 | What methodological orientation was stated to underpin the study? e.g. grounded theory, discourse analysis, ethnography, phenomenology, content analysis |  |
|  |  |  | 10 |
|  |  |  |  |
| *Participant selection* | | | |
| Sampling | 10 | How were participants selected? e.g. purposive, convenience, consecutive, snowball |  |
|  |  |  | 8-9 |
|  |  |  |  |
| Method of approach | 11 | How were participants approached? e.g. face-to-face, telephone, mail, email |  |
|  |  |  | 9 |
|  |  |  |  |
| Sample size | 12 | How many participants were in the study? | 14 |
| Non-participation | 13 | How many people refused to participate or dropped out? Reasons? | 9 |
| *Setting* | | | |
| Setting of data collection | 14 | Where was the data collected? e.g. home, clinic, workplace | 9 |
| Presence of non-  participants | 15 | Was anyone else present besides the participants and researchers? |  |
|  |  |  | 9 |
|  |  |  |  |

| Description of sample | 16 | | | What are the important characteristics of the sample? e.g. demographic data, date | |  |
| --- | --- | --- | --- | --- | --- | --- |
|  |  |  |  |  |  | Table S2 |
|  |  |  |  |  |  |  |
| *Data collection* | | | | | | |
| Interview guide | 17 | | | Were questions, prompts, guides provided by the authors? Was it pilot tested? | | Appendix |
|  |  |  |  |  |  |  |
| Repeat interviews | 18 | | | Were repeat interviews carried out? If yes, how many? | | 9 |
| Audio/visual recording | 19 | | | Did the research use audio or visual recording to collect the data? | | 9 |
| Field notes | 20 | | | Were ﬁeld notes made during and/or after the interview or focus group? | | N/A |
| Duration | 21 | | | What was the duration of the interviews or focus group? | | 9 |
| Data saturation | 22 | | | Was data saturation discussed? | | N/A |
| Transcripts returned | 23 | | | Were transcripts returned to participants for comment and/or correction? | | N/A |
| **Domain 3: analysis and**  **ﬁndings** | | | | | | |
| *Data analysis* | | | | | | |
| Number of data coders | | 24 | How many data coders coded the data? | | 8 | |
| Description of the coding  tree | | 25 | Did authors provide a description of the coding tree? | |  | |
|  |  |  |  |  | Appendix | |
|  |  |  |  |  |  | |
| Derivation of themes | | 26 | Were themes identiﬁed in advance or derived from the data? | | 8 | |
| Software | | 27 | What software, if applicable, was used to manage the data? | | 8 | |
| Participant checking | | 28 | Did participants provide feedback on the ﬁndings? | | N/A | |
| *Reporting* | | | | | | |
| Quotations presented | | 29 | Were participant quotations presented to illustrate the themes/ﬁndings?  Was each quotation identiﬁed? e.g. participant number | |  | |
|  |  |  |  |  | Table 4 | |
|  |  |  |  |  |  | |
| Data and ﬁndings consistent | | 30 | Was there consistency between the data presented and the ﬁndings? | | 10-13 | |
| Clarity of major themes | | 31 | Were major themes clearly presented in the ﬁndings? | | Table 4 | |
| Clarity of minor themes | | 32 | Is there a description of diverse cases or discussion of minor themes? | | N/A | |

**Table S3:** Measured blood pressure, GPCOG scores, HHIE-S scores and pure tone audiometry results from study cohort

| **Measured parameter** | **Total (n=35)** | **Male (n=17)** | **Female (n=18)** |
| --- | --- | --- | --- |
| Systolic BP, mmHg | 125 ± 16 | 124 ± 18 | 125 ± 15 |
| Diastolic BP, mmHg | 69 ± 8 | 69 ± 6 | 69 ±10 |
| Heart rate, bpm | 68 ± 11 | 64 ±11 | 72 ± 10 |
| GPCOG scores^1^ (median, range) | 9 (5 – 9) | 9 (5 – 9) | 9 (6 – 9) |
| HHIE-S scores^2^ (median, range) | 6 (0 – 36) | 6 (0 – 36) | 10 (0 – 28) |
| 4FA left ear^3^, dB HL | 16 ± 12 | 14 ± 11 | 17 ± 13 |
| 4FA right ear^3^, dB HL | 22 ± 14 | 21 ± 13 | 24 ± 15 |

BP: blood pressure; dB: decibels; GPCOG: General Practitioner Assessment of Cognition; HHIE-S: Hearing Handicap Inventory for Elderly – Screening; HL: hearing level; 4FA: average hearing threshold across four frequencies 500 Hz, 1 kHz, 2 kHz, 4 kHz

^1^ GPCOG scores were out of a maximum of 9 points. A score of 9 indicates no cognitive impairment; a score between 5 to 8 indicates possible cognitive impairment and monitoring is recommended; a score below 5 indicates cognitive impairment is highly likely and further investigation is recommended.
^2^ HHIE-S scores are out of 40. A score > 10 may indicate hearing impairment.
^3^ 4FA: averaged hearing threshold across 500 Hz, 1 kHz, 2 kHz, and 4 kHz

**Table S4:** Prevalence of concomitant conditions of raised blood pressure or hypertension, atrial fibrillation, possible cognitive impairment and hearing loss

| **Concomitant conditions** | **Total**  **(n=35)**  **n(%)** | **Male (n=17)**  **n(%)** | **Female (n=18)**  **n(%)** |
| --- | --- | --- | --- |
| Raised BP or hypertension^1^ and possible CI | 5(14) | 3(18) | 2(11) |
| Raised BP or hypertension and hearing loss | 8(23) | 4(24) | 4(22) |
| AF^2^ and possible cognitive impairment | 1(3) | 1(6) | 0(0) |
| Hearing loss and possible cognitive impairment | 1(3) | 1(6) | 0(0) |
| Raised BP or hypertension, AF and possible CI | 2(6) | 1(6) | 1(6) |
| Raised BP or hypertension, possible CI and hearing loss | 2(6) | 1(6) | 1(6) |

AF: atrial fibrillation; BP: blood pressure; CI: cognitive impairment

^1^ Hypertension defined as self-reported hypertension
^2^ AF includes self-reported AF and possible AF as detected from screening

**References**

1.	Stergiou GS, Tzamouranis D, Protogerou A, Nasothimiou E, Kapralos C. Validation of the Microlife Watch BP Office professional device for office blood pressure measurement according to the International protocol. *Blood Press Monit*. 2008;13(5):299-303. doi:10.1097/MBP.0b013e3283057af6

2.	Wiesel J, Fitzig L, Herschman Y, Messineo FC. Detection of Atrial Fibrillation Using a Modified Microlife Blood Pressure Monitor. *American Journal of Hypertension*. 2009;22(8):848-852. doi:10.1038/ajh.2009.98

3.	GPCOG | Home. Accessed October 22, 2024. https://gpcog.com.au/

4.	Brodaty H, Pond D, Kemp NM, et al. The GPCOG: A New Screening Test for Dementia Designed for General Practice. *J American Geriatrics Society*. 2002;50(3):530-534. doi:10.1046/j.1532-5415.2002.50122.x

5.	Kiely KM, Anstey KJ. Putting age-related hearing loss on the public health agenda in Australia. *Public Health Res Pract*. 2021;31(5):3152125. doi:10.17061/phrp3152125
